# Official Health Bulletin No. 7 of Pennsylvania State Board of Health

**Published:** 1889-09

**Authors:** 


Official Health Bulletin, No. 7, of Pennsylvania State Board of Health, is issued.

It announces a high standard of health in the Conemaugh Valley at Johnstown, the scene of the great flood.

We quote from the *Bulletin:*

"Since the issue of the last bulletin the only departure from the remarkably high standard of health which marked the period comprising the first three weeks after the flood in this valley has been a tendency to diarrhœal affections, often assuming the form of cholera morbus, less frequently than that of mild dysentery.

"This is attributable to three causes—first, the intense heat which has succeeded the unusually low temperature of the month of June; secondly, the too free use of fruits and vegetables, often unripe or over-ripe, after the enforced rigid diet which prevailed immediately after the disaster, coupled with unusual exposure to the sun's rays during the day and to dampness at night; thirdly, to the immoderate indulgence in intoxicating liquors which has prevailed since the removal of the judicial injunction on the sale of these beverages. Johnstown, however, is not the only place in the country where this tendency to bowel disorders has manifested itself, and it must be said that they have here generally yielded readily to appropriate treatment."

